# Low dose triptolide reverses chemoresistance in adult acute lymphoblastic leukemia cells via reactive oxygen species generation and DNA damage response disruption

**DOI:** 10.18632/oncotarget.13454

**Published:** 2016-11-19

**Authors:** Haijun Zhao, Pengcheng Shi, Manman Deng, Zhiwu Jiang, Yin Li, Vinodh Kannappan, Weiguang Wang, Peng Li, Bing Xu

**Affiliations:** ^1^ Department of Hematology, Nanfang Hospital, Southern Medical University, Guangzhou, P. R. China; ^2^ Department of Hematology, The First Affiliated Hospital of Xiamen University, Xiamen, P. R. China; ^3^ Guangzhou Institutes of Biomedicine and Health, Chinese Academy of Sciences, Guangzhou, P. R. China; ^4^ Research Institute in Healthcare Science, Faculty of Science and Engineering, University of Wolverhampton, Wolverhampton, UK; ^5^ Department of Hematology, Anqing Municipal Hospital of Anhui Medical University, Anqing, P. R. China

**Keywords:** triptolide, chemotherapy, drug resistance, DNA damage, acute lymphoblastic leukemia

## Abstract

Chemoresistance represents a major challenge for treatment of acute lymphoblastic leukemia (ALL). Thus, new drugs to overcome chemoresistance in ALL are urgently needed. To this end, we established a cytarabine (araC)-resistant ALL cell line (NALM-6/R), which interestingly displayed cross-resistance towards doxorubicin (ADM). Here we report that low dose of triptolide (TPL), a natural product used for treating inflammatory diseases such as arthritis, could reverse araC and ADM resistance and in NALM-6/R cells as well as primary cells from patients with relapsed or refractory (R/R) ALL, reflected by inhibition of cell proliferation and induction of apoptosis *in vitro*, and repression of tumor growth *in vivo* in a mouse xenograft model. Mechanistically, these events were associated with impaired mitochondrial membrane potential and increased reactive oxygen species (ROS) production. Co-treatment with TPL and araC or ADM upregulated pro-apoptotic caspase-9 protein, inhibited checkpoint kinase 1 (Chk1) and 2 (Chk2) phosphorylation, and induced γH2A.X (a DNA damage marker). Notably, the combination regimen of TPL and conventional chemotherapeutics also rapidly diminished tumor burden in a patient with R/R ALL. Together, these findings provide preclinical evidence for repurposing use of TPL in combination with chemotherapeutic agents to treat R/R ALL as an alternative salvage regimen.

## INTRODUCTION

Although the remission rate has achieved > 80% in patients with newly-diagnosed adult acute lymphoblastic leukemia (ALL) with standard induction regimens, a majority of the responding patients eventually become refractory to initial therapy [1, 2]. 30-60% of these patients experience a relapse, despite aggressive chemotherapy regimens for consolidation and maintenance, even after allogeneic stem cell transplantation [3]. The outcome of patients with relapsed or refractory (R/R) adult ALL remains very poor. Whereas disease-free survival and complete remission of these patients are rare after salvage therapy, most of them die from the original disease [4]. Current chemotherapy for treating ALL involves complex regimens of multiple drugs that are carefully molded in order to eliminate minimal residual disease while spare normal hematopoiesis. However, *de novo* and acquired multidrug resistance of ALL cells represents the major barrier to the success of chemotherapy. Therefore, discovery and development of new drugs to overcome multidrug resistance is urgently needed in treatment of R/R ALL patients.

Natural products, particularly those used for a long time in traditional Chinese medicine, have recently attracted a lot of attention in treatment of cancer, especially in reversing multidrug resistance [6]. Triptolide (TPL) is a diterpenoid epoxide, originally purified from the medicinal plant *Tripterygium wilfordii* Hook F (commonly known as *lei gong teng*) whose extracts have been used to treat a variety of diseases such as inflammation and arthritis in traditional Chinese medicine. TPL was structurally characterized in 1972 [5], and has been shown to have anti-inflammatory, immunosuppressive, as well as anti-cancer activity. Recently, several groups including ours have demonstrated the potential benefit of TPL to overcome chemoresistance in different types of cancer [7-9], such as myeloid leukemia, pancreatic and ovarian cancer. However, the role and underlying mechanism of TPL in reversing chemoresistance in ALL have not been explored yet.

In the present study, we first established an araC-resistant ALL cell line (NALM-6/R) by exposure of parental drug-naïve NALM-6 cells to stepwise increasing concentrations of cytarabine (araC), which also displayed cross-resistance towards doxorubicin (ADM). We then found that low dose TPL was able to re-sensitize NALM-6/R cells to araC as well as doxorubicin(ADM) *in vitro* and *in vivo*, in association with production of reactive oxygen species (ROS) and inhibition of checkpoint kinase 1 (Chk1) and 2 (Chk2), resulting in DNA damage, mitochondrial injury, and apoptosis. The regimens combining TPL with araC or ADM were highly active against primary cells obtained from patients with R/R ALL. Of note, we also observed that treatment with TPL with conventional chemotherapeutics also rapidly reduced tumor burden in a patient with R/R ALL, without notable toxicity.

## RESULTS

### Establishment of a human ALL cell line acquired chemoresistance

We first established a drug-resistant cell line (designated NALM-6/R) by exposure of the human ALL NALM-6 cells to stepwise increasing concentrations of araC, after which the established NALM-6/R cell line was routinely maintained in the medium containing 5 μM araC. The chemoresistant profile was evaluated by examine cytotoxicity of araC and other conventional anti-leukemia agents *in vitro* in parental drug-naïve NALM-6 and -resistant NALM-6/R cells. As shown in Table 1, the IC_50_ of araC against NALM-6/R cells (115.00 ± 23.12 μM) was 766 folds higher than that for parental NALM-6 cells (0.15 ± 0.07 μM; *p*< 0.01). Interestingly, NALM-6/R cells displayed marked cross-resistance towards ADM, with the IC_50_ of ADM in NALM-6/R cells (4.82 ± 0.97 μM) about 43 folds higher than that in parental NALM-6 cells (0.11 ± 0.03 μM; *p*< 0.01). Of note, there was no cross-resistance to TPL observed in NALM-6/R cells (IC_50_ = 0.032 ± 0.04 μM vs. 0.035 ± 0.03 μM for parental NALM-6 cells; *p*> 0.05). These results demonstrate that NALM-6/R cells, a chemoresistant human ALL cell line established *in vitro*, are highly tolerated to cytotoxicity of conventional chemotherapeutic agents such as araC and ADM.

**Table 1:** Cytotoxicity of TPL, araC ADM against drug-naïve NALM-6 cells and their chemoresistant NALM-6/R counterparts

Agents	IC_50_ (μM)		Fold increase	*P* value
	NALM-6	NALM-6/R		
TPL	0.035 ± 0.003	0.032 ± 0.004	0.91	> 0.05
araC	0.15 ± 0.07	115.00 ± 23.12	766.67	< 0.01
ADM	0.11 ± 0.03	4.82 ± 0.97	43.82	< 0.01

Note: Cytotoxicity was assessed using the CCK-8 assay.

### Low dose TPL enhances cytotoxicity of araC and ADM in NALM-6/R cells

**Figure 1:**
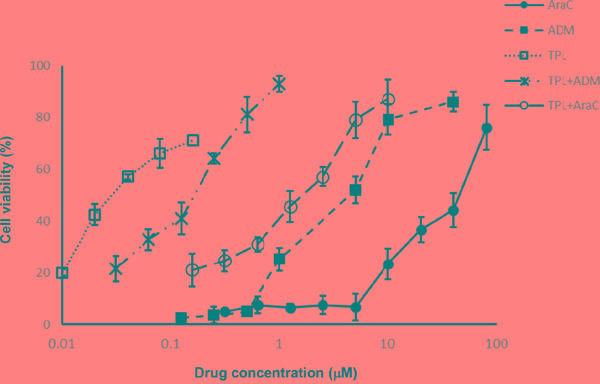
TPL re-sensitizes chemoresistant NALM-6/R cells to araC and ADM NALM-6/R cells were exposed to the indicated concentrations of araC or ADM ± TPL (IC_20_: 10 nM) for 48 h, after which cell viabilities were determined by a CCK-8 assay. Values indicate mean ± SD for three independent experiments.

The inhibitory effect of TPL, araC and ADM as single agent on growth of NALM-6/R cells was first examined. Neither araC within a concentration range of 0-5 μM nor ADM within a concentration range of 0-0.5 μM exhibited significant proliferation inhibition in NALM-6/R cells after 48h exposure (Figure 1). However, in the presence of the IC_20_ concentration (i.e., 10 nM) of TPL, the IC_50_ of araC and ADM against NALM-6/R cells were reduced by 20 and 15 times, respectively (Table 2 and Figure 1). These results suggest that low dose TPL might be capable to reverse chemoresistance against araC and ADM in ALL cells.

**Table 2:** TPL-enhanced cytotoxicity of araC or ADM in NALM-6/R cells

Agents	IC_50_ (μM)	Fold decrease	*P* value
AraC	45 ± 3.27	−	< 0.01
AraC (+ TPL*)	2.25 ± 0.72	20.00	
ADM	4.85 ± 0.95	−	< 0.01
ADM (+ TPL*)	0.32 ± 0.081	15.16	

Note: Cytotoxicity was assessed using the CCK-8 assay. * TPL = 10 nM (IC_20_ concentration).

### TPL potentiates araC- or ADM-induced apoptosis in both NALM-6/R cells and primary refractory or relapsed ALL cells

**Figure2:**
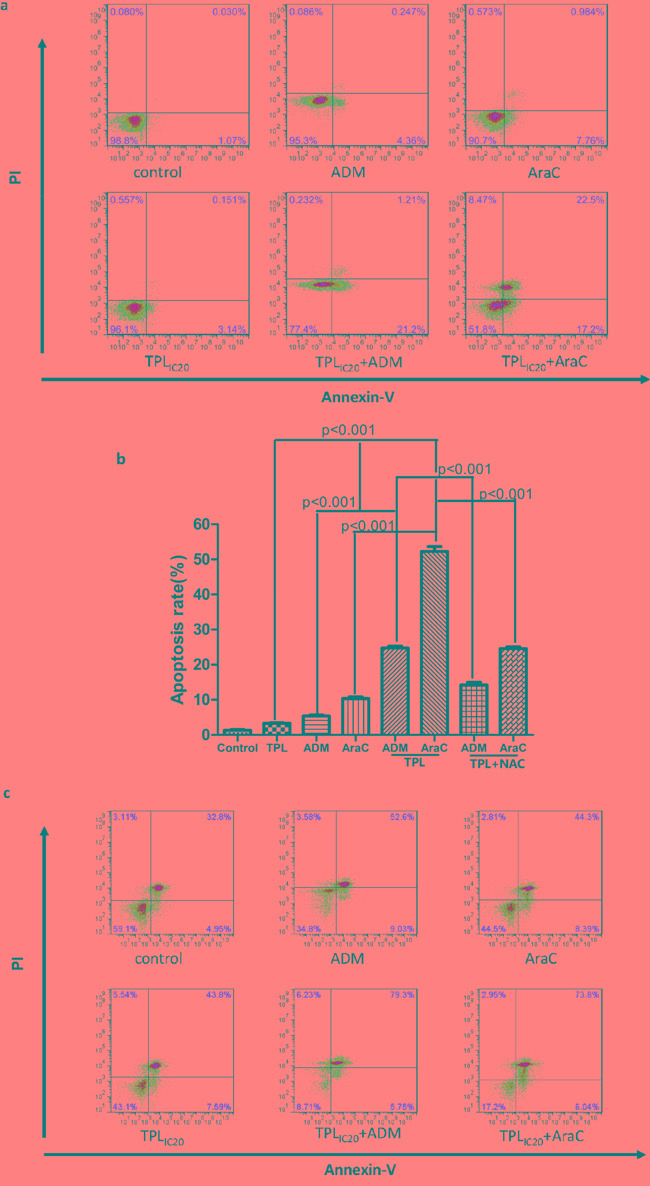
TPL increased apoptosis induced by araC or ADM in NALM-6/R cells, an event blocked by the antioxidant NAC, as well as in primary R/R ALL cells **a.** NALM-6/R cells were exposed to araC (5 μM) or ADM (0.5 μM) ± TPL (IC_20_: 10 nM) for 48 h, after which apoptotic ratios were determined by Annexin V/PI staining and flow cytometry. **b.** A column chart summarizes apoptotic ratios of NAML/R cells after drug treatments as described above for at least three independent experiments (mean ± SD). In addition, 30 mM NAC was added for 2 h to block ROS generation prior to combined treatment with TPL plus araC or ADM. **c.** Primary cells isolated from a patient with R/R ALL (patient #1 as shown in Table 3) were incubated with araC (5 μM) or ADM (0.5 μM) ± TPL (IC_20_: 10 nM) for 48 h, after which apoptosis was analyzed Annexin V/PI staining and flow cytometry.

We then investigated whether the synergistic anti-tumor effects between triptolide and araC or ADM stem from induction of apoptosis in chemoresistant ALL cells. To this end, NALM-6/R cells were exposed to the highest non-cytotoxic concentrations of araC (5 μM) or ADM (0.5 μM) in the presence or absence of low dose (IC_20_ concentration, 10 nM) TPL for 48 h. Notably, co-administration of low dose TPL significantly increased apoptosis induced by araC or ADM from 10.21 ± 0.07% and 5.56 ± 0.04% to 52.40 ± 4.45% and 24.60 ± 3.23% (*P* < 0.01 for each case; Figure 2a and 2b), respectively. Consistently, combined treatment with 10 nM TPL and sub-toxic concentrations of araC or ADM significantly increased apoptosis in primary cells isolated from R/R ALL patients (n = 12; *P* < 0.01 for each case, compared araC or ADM as single agent; Figure 2c and Table 3). Notably, the regimens combining TPL with araC or ADM were more effective to induce apoptosis in primary R/R B-ALL cells from patients with white blood cell counts > 100 x 10^9^/L than those with < 100 x 10^9^/L (*P* < 0.05; Table 4). These findings suggest that TPL might re-sensitize chemoresistant ALL cells to araC or ADM.

**Table 3:** Effects of the regimens combining TPL with araC or ADM on primary blast cells of refractory or relapsed B-ALL patients (n = 12)

No	Age/sex	Disease status	Cytogenetic	Prior therapy	Apoptotic cells (%)					
					Con	TPL*	araC	ADM	araC+TPL*	ADM+TPL*
1	43/M	Refractory	t(9;22)	TKIs, VDLP; Hyper-CVAD; Allo-SCT	32.71±3.13	34.32±3.65	37.28±5.70	36.53±2.65	58.46±8.78	51.47±10.16
2	65/F	Refractory	Complex	VDCLP; Hyper-CVAD	38.70±4.28	54.84±3.25	52.76±4.89	62.31±5.17	81.76±3.42	89.27±4.33
3	19/F	Relapse 2	Complex	VDLP; Hyper-CVAD;FA	34.68±8.78	38.32±7.64	43.73±5.41	56.75±3.73	59.79±3.51	67.32±8.38
4	35/M	Refractory	t(9;11)	VDCLP; Hyper-CVAD;FA	24.71±6.62	31.32±6.22	36.28±4.37	42.62±8.19	55.26±7.43	52.15±8.54
5	43/F	Refractory	t(5;14)	VDCLP; Hyper-CVAD	27.52±7.14	32.25±2.67	38.78±3.69	36.62±10.3	54.52±4.71	48.25±3.16
6	62/M	Relapse	t(9;22)	TKIs, VDLP	32.56±5.76	35.24±3.32	35.35±5.18	37.22±4.87	58.29±4.68	51.61±5.39
7	64/F	Relapse 2	Hypodiploidy	VDCLP; CAM; MA; Hyper-CVAD	36.28±6.29	39.11±2.67	42.68±3.72	45.51±5.62	60.37±4.31	54.49±7.48
8	17/F	Refractory	Complex	VDLP; Hyper-CVAD; VDCLP	27.40±8.13	31.28±3.42	34.63±5.75	32.48±4.22	44.32±8.78	36.71±4.95
9	37/M	Relapse	t(4;11)	VDCLP; CAM	26.52±3.68	32.61±7.73	37.31±6.15	30.22±5.34	42.86±6.38	34.47±9.59
10	26/F	Refractory	t(9;22)	TKIs, VDLP; Hyper-CVAD	31.74±4.23	36.56±3.46	38.51±5.76	38.25±6.48	48.42±6.73	41.67±5.98
11	59/M	Refractory	Complex	VDCLP; CAM;MA	36.86±4.37	36.17±2.36	41.51±3.45	40.69±5.78	46.87±6.22	44.54±5.34
12	28/F	Relapse 2	Complex	VDCLP; CAM;MA; Hyper-CVAD	24.73±3.74	31.28±4.38	34.21±3.67	37.27±5.38	39.18±6.23	44.02±7.43

Note: Percentage of apoptotic cells was determined by Annexin V/PI staining and flow cytometry. Values indicate mean ± SD for at least triplicate experiments. *TPL = 10 nM (IC_20_ concentration).

Relapse 2, patients with second relapse; Allo-SCT, allogeneic stem cell transplant; TKIs, tyrosine kinase inhibitors (imatinib or dasatinib; VD(C) LP: vincristine, daunorubicin, (cyclophosphamide), L-Asparaginase and prednisone; Hyper-CVAD: hyper-fractionated cyclophosphamide, vincristine, doxorubicin, and dexamethasone, alternating with high-dose methotrexate and cytarabine; FA: fludarabine and cytarabine; MA: mitoxantrone and cytarabine; CAM: cyclophosphamide, cytarabine, and 6-mercaptopurine.

**Table 4:** The relationship between WBC count and cytotoxicity of the regimens combining TPL with araC or ADM in primary refractory or relapsed B-ALL cells

WBC at biopsy (x10^9^/L)		Con	TPL*	araC	ADM	araC+TPL*	ADM+TPL*	*P* value
Median	114							
Range	23-146							
>100	n = 7	33.74±4.83	39.37±8.25	42.87±6.46	45.85±11.52	61.36±10.12	59.67±15.12	< .05
<100	n = 5	30.93±4.23	35.01±4.23	38.07±3.27	37.15±4.04	42.52±3.61	40.08±5.72	
Total	n = 12	32.57±4.62	37.56±6.98	40.87±5.73	42.23±9.92	53.51±12.44	51.50±15.44	< .01

Note: Percentage of apoptotic cells was determined by Annexin V/PI staining and flow cytometry. Values indicate mean ± SD for the indicated number of patients. *TPL = 10 nM (IC_20_ concentration).

### The combination of araC or ADM with TPL triggers reactive oxygen species (ROS) production and induces mitochondrial injury in ALL cells

**Figure 3:**
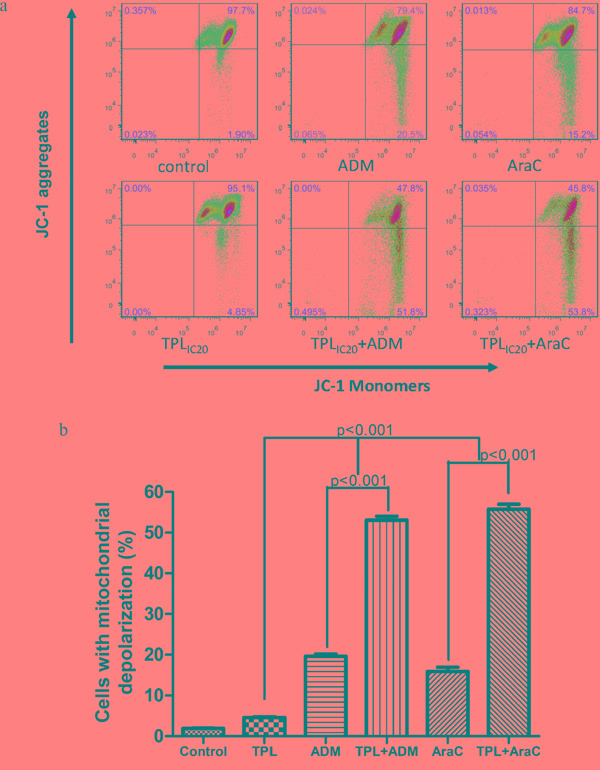
Combined treatment with TPL and araC or ADM results in mitochondrial injury in NALM-6/R cells **a.** NALM-6/R cells were exposed to araC (5 μM) or ADM (0.5 μM) ± TPL (IC_20_: 10 nM) for 48 h, after which mitochondrial membrane potential (ΨΔm) was determined by monitoring JC-1 aggregates using flow cytometry. **b.** A column chart summarizes percentages of NAML/R cells with loss of ΨΔm (or mitochondrial membrane depolarization) after drug treatments as described above for at least three separate assays (mean ± SD).

Since mitochondria play a crucial role in regulation of apoptosis, apoptosis is often associated with loss of mitochondrial membrane potential (MMP) [10]. In this context, we then examine the effects of TPL and araC or ADM alone or in combination on MMP. As shown in Figure 3, whereas exposure to araC (5 μM) or ADM (0.5 μM) resulted in a modest decrease in JC-1 aggregates, co-administration of 10 nM TPL with either of these agents sharply reduced JC-1 aggregates (Figure 3a), reflecting loss of MMP (or mitochondrial depolarization), in NALM-6/R cells (Figure 3b, *P* < 0.001 compared to each agent alone).

**Figure 4:**
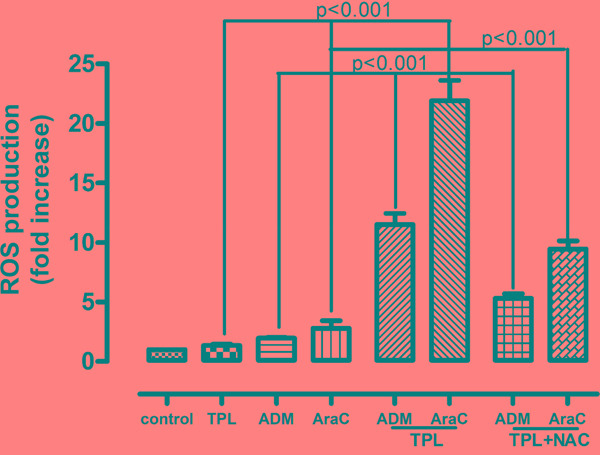
Combined treatment with TPL and araC or ADM induce ROS production in NALM-6/R cells, an event prevented by NAC NALM-6/R cells were exposed to araC (5 μM) or ADM (0.5 μM) ± TPL (IC_20_: 10 nM) for 12 h, as well as addition of 30 mM NAC for 2 h prior to combined treatments, after which ROS was measured using H_2_DCFDA dye and flow cytometry. Values indicate fold increases, compared to untreated control, for three separate experiments (mean ± SD).

Moreover, considering the important role of ROS in depolarizing mitochondria and inducing apoptosis, we further measured the ROS levels in NALM-6/R cells after exposed to araC (5 μM) or ADM (0.5 μM) ± 10 nM TPL for 12h. Compared to treatment with each single agent, the combination of TPL with either araC or ADM significantly increased ROS generation by approximately 9 and 5 folds in NALM-6/R cells, respectively. Notably, 2h pre-treatment with the ROS scavenger NAC (30 mM) dramatically prevented ROS production induced by TPL plus araC or ADM (Figure 4), resulting in a marked reduction in apoptosis (from 52.40 ± 4.45% and 24.60 ± 3.23% to 24.56 ± 3.17% and 14.15 ± 2.41%, respectively) in NALM-6/R cells (Figure 2b, *P* < 0.001). These findings indicate that TPL potentiates lethality of araC and ADM in chemoresistant ALL cells likely by inducing ROS generation and mitochondrial injury.

### Combined treatment with TPL and araC or ADM disrupts DNA damage checkpoint, resulting in robust DNA damage in NALM-6/R cells

**Figure 5:**
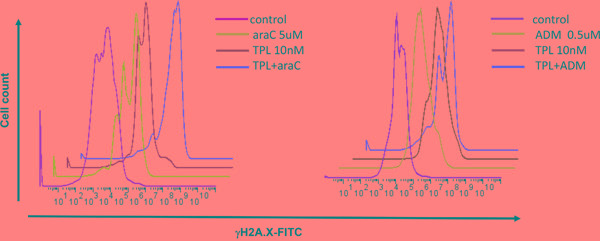
Co-exposure to TPL and araC or ADM leads to increased expression of γH2A.X, a marker for DNA double-strand break NALM-6/R cells were exposed to araC (5 μM, left panel) or ADM (0.5 μM, right panel) ± TPL (IC_20_: 10 nM) for 48 h, after which cells were stained with anti-γH2A.X antibody for 1 h, followed by FITC-conjugated secondary antibody for 30 min, and then subjected to flow cytometric analysis.

Both AraC and ADM, known as DNA cross-linking agents, act to induce DNA damage in cancer cells [11, 12], manifested by increased S139 phosphorylation of histone H2A.X (designated γH2A.X) at the sites of DNA double-strand break [13]. As γH2A.X is commonly used as a marker for DNA damage, we next examined expression of γH2A.X to monitor the effects of TPL on DNA damage induced by araC or ADM. While TPL did not significantly affected the levels of γH2A.X, the combination of TPL with araC or ADM at the indicated doses resulted in a rightward shift of γH2A.X-FITC fluorescent peak in FACS histograms, indicating increased γH2A.X expression (Figure 5).

**Figure 6:**
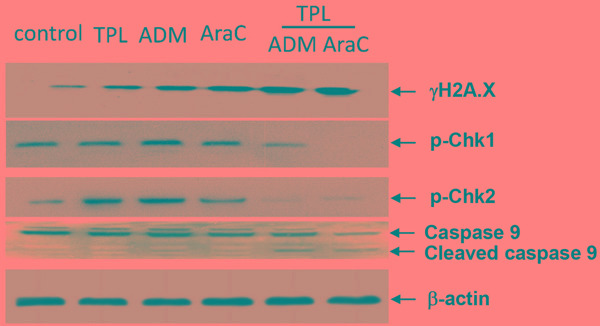
Co-treatment with TPL and araC or ADM disrupts DNA damage checkpoint and induces caspase 9 activation in NALM-6/R cells NALM-6/R cells were exposed to araC (5 μM) or ADM (0.5 μM) ± TPL (IC_20_: 10 nM) for 48 h, after which Western blotting analysis was performed to monitor expression of γH2AX, p-Chk1, and pChk2, as well as cleavage/activation of caspase 9. The representative blots are shown for three independent experiments.

Further, Western blot was performed to assess the effects of TPL and araC or ADM alone or in combination on DNA damage checkpoint by monitoring phosphorylation of Chk1 (Ser345) and Chk2 (Thr68), which reflects cytoprotective activation of cell cycle checkpoints in response to DNA damage (e.g., induced by DNA-damaging agents, including araC and ADM) [14]. Notably, co-treatment with TPL markedly diminished phosphorylation of Chk1 and/or Chk2 triggered by araC and ADM, accompanied by a robust increase in γH2A.X expression (Figure 6), consistent with the results of flow cytometric analysis (Figure 5), and cleavage/activation of caspase 9. These findings suggest that disruption of DNA damage checkpoint might also contribute to reversal of chemoresistance by TPL in ALL cells.

### The regimen combining TPL and araC is active *in vivo* in a xenograft mouse model generated from chemoresistant ALL cells

**Figure 7:**
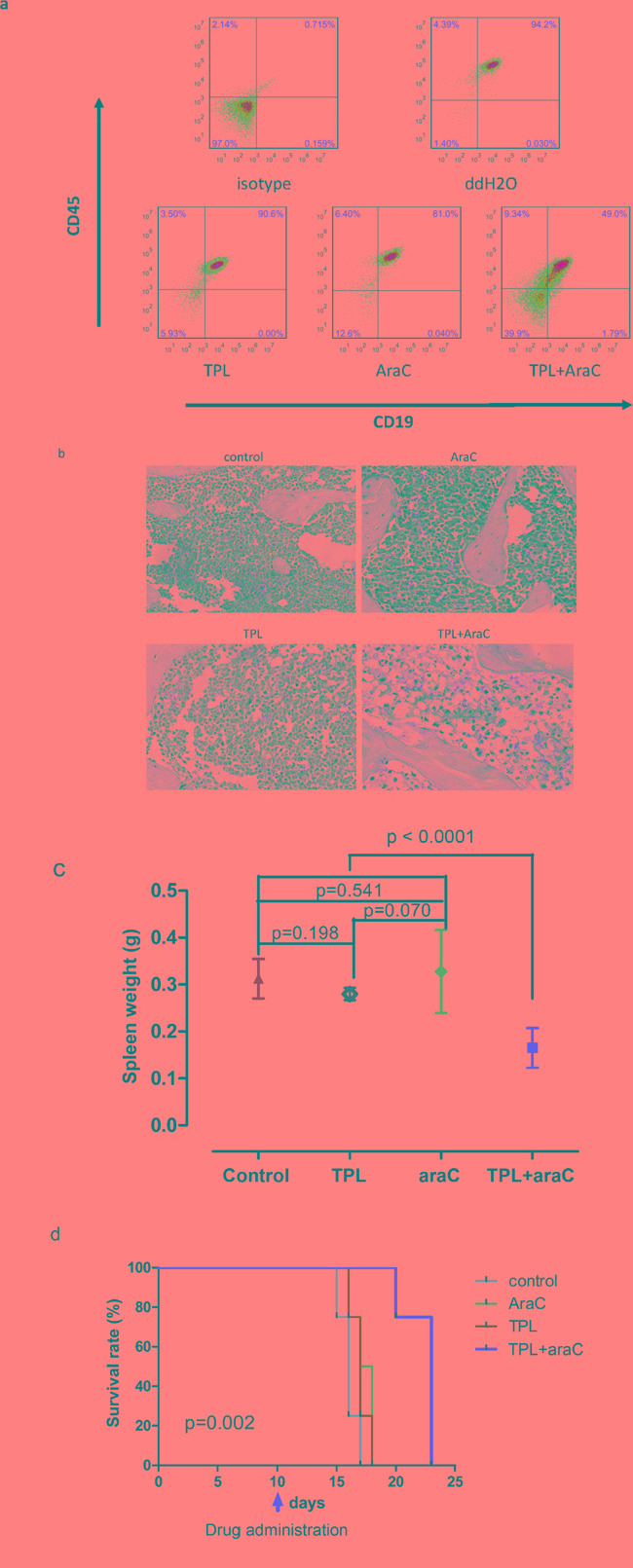
The regimen combining TPL with araC reduces tumor burden and prolongs animal survival in a mouse xenograft model of NALM-6/R cells 5 × 10^5^ NALM-6/R cells were subcutaneously injected via angular veins into sublethally irradiated adult NSI (NOD/SCID IL2rg-/-) mice. After 10 days, tumor-bearing mice were given intraperitoneally for 5 days with 100 μl ddH_2_O as control, 10 mg/kg araC, 0.5 mg/kg TPL, or TPL in combination with araC, respectively. On day 16 post tumor cell inoculation, flow cytometry was performed to monitor the number of human CD45/CD19 double-positive cells in bone marrow **a.**; bone marrow sections were stained with H & E staining **b.**; spleen weight was measured **c.** Kaplan-Meier analysis was performed to assess animal survival **d.**

To validate the anti-leukemia activity of the regimen combining TPL and araC *in vivo*, we established a xenograft mouse model by intravenous injection with chemoresistant NALM-6/R cells into NSI (NOD-SCID-IL2Rg-/-) mice. Of note, mice treated with TPL plus araC showed a substantial reduction of tumor burden, manifested by a marked decrease inCD45/CD19 double-positive cells in bone marrow, compared to mice receiving each single agent (Figure 7a). Consistently, histopathology revealed a remarkable reduction of leukemia cell infiltration in bone marrow of mice receiving treatment with TPL plus araC (Figure 7b). Further, average spleen weight of mice treated with TPL plus araC (0.17 ± 0.26 g) were significantly lower than those of mice in the control group (0.31 ± 0.23 g), as well as each single agent group (0.28 ± 0.01g for TPL alone and 0.33 ± 0.56 g for araC alone; *P* <0.0001 for each case; Figure 7c). Last, Kaplan-Meier analysis showed that the combination of TPL with araC significantly prolonged animal survival, compared to TPL or araC alone (*P* < 0.01, Figure 7d).

### The combined therapy with TPL and chemotherapeutics rapidly reduces tumor burden in a patient with refractory B-ALL

Finally, we report the first use of TPL in combination with conventional chemotherapeutic agents to treat one patient with refractory ALL (patient #2, Table 3), who had no other treatment available when enrolled into this study. This pilot study was conducted with an approval from the Departmental Review Board and the patent's informed consent. Data was collected prospectively. The patient was a 65-year-old female diagnosed with pre B-ALL with complex chromosome abnormalities. Even though two courses of induction chemotherapy including VDCLP and hyper-CVAD/A regimens were administrated, the response assessments unfortunately indicated a minor remission. At enrollment, the patient suffered from severe bone pain and fatigue; the complete blood count (CBC) revealed WBC count of 145×10^9^/L; a high percentage of blasts (60 and 86%, respectively) were detected on the smears of both peripheral blood (PB) and bone marrow (BM) aspirate; other laboratory tests included a basic metabolic panel, liver and coagulopathy panels, and renal function, which were all unremarkable. Considering the refractory status of the disease together with the results of the clinical tests, TPL in combination with the FA (fludarabine and cytarabine) regimen was then administered as salvage chemotherapy. The dose level of TPL was 100 μg/m^2^ administered in 3 divided doses daily for 7 days, which was routinely used in clinic for treatment of rheumatoid arthritis [15]. TPL-related hematological or extra-hematological toxicities, including the most serious side effects of TPL such as renal toxicity, were not observed during the course of treatment. Surprisingly, the patient experienced marked hematological improvement with an 80% reduction of peripheral blood blasts two weeks after treatment, and complete resolution of bone pain and fatigue. However, the prolonged response to TPL-associated treatment remains to be defined.

## DISCUSSION

Development of salvage regimens for adult patients with ALL has focused on the incorporation of alternative agents with their known roles in this disease. Most of the recently developed regimens involve the use of high-doses of araC in combination with various other agents [16-19]. However, efficacy of these regimens is significantly hampered by acquired drug-resistance to araC. Therefore, reversal of araC resistance might improve the clinical outcomes of ALL. To this end, we established an araC-resistant ALL cell line (NALM-6/R), which unexpectedly was also cross-resistant to ADM. Of note, low dose TPL (10 nM) was able to increase the sensitivity of NALM-6/R cells to araC and ADM by 20 and 15 times, respectively. Further, low dose TPL also significantly increased apoptosis induced by araC and ADM in NALM-6/R cells. We further established a xenograft mouse model using these araC-resistant NALM-6/R cells, in which low dose TPL plus araC was also highly active *in vivo*, reflected by marked reduction of tumor burden and prolonged animal survival.

Importantly, low dose TPL also dramatically enhanced lethality of araC or ADM in primary leukemia blasts isolated from patients with R/RALL despite the cytogenetic subtypes. It is well established that disease burden at time of relapse is one of the most important prognostic factors of R/R ALL [4]. Interestingly, combined treatment with TPL and araC or ADM induced higher percentage of apoptosis in blasts of patients with WBC count > 100×10^9^/L, than those with < 100×10^9^/L, suggesting that this combination regimen might overcome the unfavorable effects of high tumor burden. However, further studies including more cases are needed to confirm this finding. Notably, after received the combination therapy of TPL with conventional chemotherapeutics, a patient with refractory ALL, who had no other treatment available, experienced hematological improvement with 80% reduction of peripheral blood blasts two weeks later. Taken together, these *in vitro* and *in vivo* findings argue strongly that low dose TPL might re-sensitize chemoresistant ALL cells to conventional chemotherapeutic agents (e.g., araC and ADM), and thereby improve the outcome of patients with R/R ALL.

Chemotherapy represents a plethora of challenges to chromosomal DNA. Whereas DNA damage caused by various stimuli has been known to increase ROS levels [20], ROS can in turn induce a wide array of damages to DNA, therefore forming a positive feedback loop. DNA damage-induced ROS is important for determination of cell death versus survival [21]. The ability of cancer cells to distinguish ROS as a pro-survival from a pro-apoptotic signal is dependent on the amount of ROS. While modest levels of ROS are required for cancer cells to survive, excessive ROS production is lethal to them. It has been reported that drug-resistant cells often have relatively higher basal levels of intrinsic oxidative stress, which might thus lower their threshold to lethal actions of ROS [22]. In the present study, we found that combined treatment with TPL and araC or ADM not only markedly increased ROS production, but also induced robust DNA damage in chemoresistantNALM-6/R cells. Moreover, pre-treatment with the antioxidant NAC attenuated ROS generation triggered by TPL plus araC or ADM, resulting in blockade of apoptosis, suggesting the functional role of elevated ROS levels in reversal of chemoresistance by TPL. Triptolide has previously been demonstrated to induce apoptosis by regulating members of the caspase family, in association with loss of MMP and activation of caspases in cancer cells [23, 24]. Notably, combined treatment with TPL and araC or ADM led to loss of MMP as well as cleavage/activation of caspase-9. Taken together, these findings argue that TPL in combination with araC or ADM triggers ROS production and induces DNA damage, cooperatively leading to activation of the mitochondria-mediated intrinsic apoptosis pathway.

Virtually all DNA-damaging agents currently used in cancer treatment induce apoptosis of cancer cells by inducing substantial DNA damage. It is well established that efficient and continual DNA repair is crucial for normal cells to survive, while it also represents an important mechanism for chemoresistance of tumor cells. Thus, defects or disruption of the DNA damage response (DDR), including DNA damage checkpoint and repair pathways, could sensitize cancer cells to genotoxic agents. For example, the Chk1 inhibitor 7-Hydroxystaurosporine (UCN-01) has been shown to potentiate anti-tumor activity of the DNA-damaging agents such as gemcitabine and cytarabine [25]. DNA cross-linking agents activate checkpoint kinase 1 (Chk1) and 2 (Chk2), which are essential transducer of the DDR [14], which protect cells from lethal effects of genotoxic stress induced by these agents. In the present study, it was found that co-administration of TPL significantly inhibited phosphorylation/activation of Chk1 and Chk2 induced by araC or ADM, accompanied by robust DNA damage and apoptosis. These results are consistent with a recent finding that TPL potentiates cisplatin-induced apoptosis by inhibiting DNA repair in lung cancer cells [26].

In summary, the present study demonstrates that low dose TPL could reverse chemoresistance of ALL cells to conventional DNA-damaging agents (e.g., araC- and ADM), at least in part through induction of ROS, disruption of the DDR, and increase of DNA damage. It also provides the preclinical and pilot clinical evidence suggesting that the potential clinical benefits of adding TPL into standard DNA-damaging agents-based chemotherapy regimens warrant attention in treatment of relapsed and refractory ALL patients.

## MATERIALS AND METHODS

### Chemicals and reagents

Triptolide (TPL, C_20_H_24_O_6_, MW: 360.40), cytarabine (araC, C_9_H_13_N_3_O_5_, MW: 243.22), doxorubicin (ADM, C_27_H_29_NO_11_, MW: 543.52), and N-acetyl-L-cysteine (NAC) were purchased from Sigma-Aldrich (St. Louis, MO). TPL was dissolved in dimethyl sulfoxide (Sigma-Aldrich, Dorset, UK) as a 100 μM stock solution, and was freshly diluted in culture medium before use. Cytarabine, doxorubicin, were dissolved in phosphate-buffered saline (PBS) as a 100 mM stock solution at -20°C. Light exposure was kept to a minimum for all drugs used.

### Cell culture and patient samples

NALM-6 cells were obtained from the Institute of Hematology and Blood Diseases Hospital, Chinese Academy of Medical Sciences (Tianjin, China). The cells were routinely cultured in a RPMI 1640 medium (Invitrogen, USA), supplemented with 10% fetal bovine serum at 37°C in a humidified 5% CO_2_ incubator under standard conditions. AraC-resistant cells were established from the parental NALM-6 cells in our laboratory by continuous exposure to araC at low but gradually increasing concentrations, as described previously [27]. Finally, NALM-6/R cells were maintained in complete medium containing 5 μM of araC.

Relapsed or refractory acute lymphoblastic leukemia (R/R ALL) cases were defined according to the classification in the NCCN guidelines. Twelve cases of R/R ALL bone marrow or peripheral blood samples were obtained from the Nanfang Hospital, Southern Medical University. Major patient characteristics are summarized in Table 3. Mononuclear cells were isolated by standard Ficoll-Hypaque density centrifugation, and then cultured in RPMI-1640 supplemented with 10% fetal bovine serum. The acquisition of human bone marrow samples were approved by the local institutes and the experimental use of human specimens was carried out in accordance with the institutional guidelines and the Declaration of Helsinki. Acquisition of bone marrow samples was performed with the informed consent of patients.

### *In vitro* cell proliferation assay

NALM-6/R cells were cultured in 96-well culture plates at a density of 5 × 10^4^ cells/well in a medium containing different concentrations of araC, ADM, or combinations of these agents with fixed concentrations of TPL (IC_20_: 10 nM for 48 h) at 37°C in a humidified 5% CO_2_-95% air incubator. Cell inhibition was determined by a CCK8 assay, according to the manufacturer's instructions (Beyotime Company, China). After 48 h, the cells were incubated in a 10 μl CCK-8 solution for 2 h at 37°C. The absorbance of each well was quantified at 450 nm with an automated ELESA reader. IC_50_ values were obtained using the logit method, and were determined from the results of at least 3 independent tests. The inhibition rate was calculated based on the following formula: Inhibition rate (%) = (1 - absorbance of experimental group/absorbance of control group) x 100%.

### Flow cytometric assays for cell apoptosis, mitochondrial membrane potential, reactive oxygen species and γH2A.X detection

After treated for 48 h, 2 x 10^5^ NALM-6/R cells were subjected to apoptosis assay using an Annexin V Apoptosis Detection Kit-APC (eBioscience Company, USA), following the manufacturer's instructions. For the mitochondrial membrane potential assessment, after different treatments for 48 h, 2 x 10^5^ NALM-6/R cells were analyzed using a JC-1 fluorescent probe kit (Beyotime Company, China), following the manufacturer's instructions. To measure the intracellular reactive oxygen species (ROS) levels, about 2 x 10^5^ NALM/R cells subjected to different treatments were washed in PBS buffer twice, and then incubated in 1 ml of serum-free RPMI 1640 medium containing 10 μM of H_2_DCFDA for 30 min at 37°C. The cells were harvested, and then washed in serum-free RPMI 1640 medium buffer twice to remove the remaining H_2_DCFDA. The fluorescent intensity was measured by flow cytometry. For assessing the degree of DNA damage, 2 x 10^5^ NALM-6/R cells were incubated for 15 min on ice in hybridization buffer (PBS containing 0.5% bovine serum albumin (BSA) and 0.25% Triton X-100). After centrifugation, the cells were incubated with rabbit monoclonal anti-γH2A.X antibody (Cell Signaling Technology, USA) for 1 h, then washed with PBS and incubated with an FITC-conjugated mouse anti-rabbit IgG antibody (BD Pharmingen) for 30 min in the dark at room temperature.

### Western blot analysis

The cytoplasmic protein (50 μg/lane) from each sample was loaded onto a 10% SDS-PAGE gel, then transferred to a PVDF membrane (Millipore, Billerica, MA, USA) and blotted with the appropriate antibodies. Non-specific binding was avoided by blocking the PVDF membrane with 5% skimmed milk in TBS-T for 1 h. The 5% skimmed milk in TBS-T was also used to dilute primary antibodies (phospho-γ-H2AX, rabbit mAb,1: 1000, CST; Phospho-Chk1 (Ser345) Rabbit mAb, detecting Chk1 phosphorylated at serine 345,1: 1000, CST; Phospho-Chk2 (Thr68) Rabbit mAb, detecting Chk2 phosphorylated at Thr68,1: 1000, CST; caspase-9, mouse mAb, 1: 1000, CST) and HRP-conjugated secondary antibody (1: 10000; CST). The membranes were incubated in the primary antibodies overnight at 4°C and in the secondary antibody for 1 h at room temperature. The quantities of protein loaded were verified by staining the same membranes with anti-β-actin antibody (rabbit mAb 1: 1000, CST). The signals were detected on X-ray films using an enhanced chemiluminescence western blotting detection kit (Amersham Pharmacia Biotech). β-Actin was included as a loading control.

### Animal study

All animal experiments were performed in the Laboratory Animal Center of the Guangzhou Institute of Biomedicine and Health (GIBH), and the animal procedures were approved by the Animal Welfare Committee of GIBH. To generate a mouse model of araC-resistant ALL, we subcutaneously injected NALM-6/R cells (5 × 10^5^) into the angular veins of sublethally irradiated adult NSI (NOD/SCID IL2rg-/-) mice (20-30 g body weight; 2-3 months of age). Ten days later, 16 tumor-bearing mice were divided into 4 groups and injected intraperitoneally with 100 μl with ddH_2_O, araC (10 mg/kg, half of maximum tolerated dose in SCID mice [28]), TPL (0.5 mg/kg, 25% of lethal concentration [29]), or TPL in combination with araC for 5 days, respectively. We evaluated the responses to this treatment by measuring the overall survival times, the weights of the spleens and the percentages of human CD45/CD19 double-positive cells in the bone marrow, as assessed by flow cytometry. The tissue samples were fixed in formaldehyde and further embedded in paraffin.

### Statistical analysis

Data was expressed as the mean ± standard deviation (S.D.) for at least three independent experiments and compared using the Student *t*-test. Multiple-group comparisons were performed using the One-way analysis of variance (ANOVA) followed by the Bonferroni posthoc test. Survival were estimated using the Kaplan-Meier analysis and compared using the log-rank test. *P* values < 0.05 were considered statistically significant. Statistical analyses were performed using SPSS 20.0 software (La Jolla, CA).
